# Treatment of Facial Basal Cell Carcinoma: A Review

**DOI:** 10.1155/2011/380371

**Published:** 2011-04-27

**Authors:** Vanessa Smith, Shernaz Walton

**Affiliations:** Department of Dermatology, Hull Royal Infirmary, Hull and Hull York Medical School (HYMS), Hull HU2 3JZ, UK

## Abstract

Basal cell carcinomas (BCCs) are locally destructive malignancies of the skin. They are the most common type of cancer in the western world. The lifetime incidence may be up to 39%. UV exposure is the most common risk factor. The majority of these tumours occur on the head and neck. Despite BCCs being relatively indolent the high incidence means that their treatment now contributes a significant and increasing workload for the health service. A good understanding of the options available is important. Management decisions may be influenced by various factors including the patient's age and comorbidities and the lesion subtype and location. Due to the importance of a good cosmetic and curative outcome for facial BCCs treatment decisions may differ significantly to those that would be made for BCCs arising elsewhere. There is little good randomized controlled data available comparing treatment modalities. Although traditionally standard excision has been the treatment of choice various other options are available including: Mohs micrographic surgery, curettage and cautery, cryosurgery, radiotherapy, topical imiquimod, photodynamic therapy and topical 5-fluorouracil. We discuss and review the literature and evidence base for the treatment options that are currently available for facial BCCs.

## 1. Introduction

Basal cell carcinomas (BCCs) are locally destructive malignancies of the skin. They are the most common type of cancer in Europe, Australia [1] and the U.S.A [2]. A Canadian study found the lifetime incidence in the Caucasian population to be between 15%–28% in women and 17%–39% in men [3]. In the U.K. the true incidence is not known due to inconsistencies in cancer registration [4]. However, estimates suggest that 53,000 new cases are diagnosed in the UK each year [5]. Despite BCCs being relatively indolent the high incidence means that the treatment of these tumours contributes a significant and ever growing workload for the NHS. 

The most significant aetiological factors appear to be exposure to ultraviolet radiation and genetic predisposition [6]. BCCs tend to occur in areas of chronic sun exposure and therefore a large proportion, around 74%, occurs on the head and neck [3]. Although BCCs are usually slow growing and rarely metastasize [7], local destruction, and disfigurement may occur if left untreated or if incompletely removed [8]. 

Management is dependent upon a variety of factors, including the location of the lesion, the patient's age, comorbidities and the type of tumour involved. The location of the lesion is important, as tumours that arise in cosmetically or functionally important areas are best managed with treatments that minimise the amount of tissue removed whilst ensuring a high chance of complete cure. In the elderly population, the slow growing nature of BCCs means that less invasive treatments may be favoured despite the fact that some of these methods have higher recurrence rates. Cystic and nodular BCCs (nBCC) have relatively well defined borders, while morphoeic, micronodular, trabecular, infiltrative and basosquamous BCCs are often less well defined and are also more aggressive [9]. Superficial BCCs (sBCC) may be amenable to topical treatments as a result of their minimal depth of invasion. 

Over recent years, various treatments beside traditional excision have been tried in an effort to provide better results, in terms of reduction of recurrence, better patient acceptability, and improved cosmesis. Although many treatments are now used for BCCs, there is little research that accurately compares these different treatment modalities against each other for different types of tumours in different locations. As a result of the importance of a good cosmetic outcome when tumours arise on the face treatment decisions may differ significantly to those that would be made for BCCs arising elsewhere. We discuss and review the literature and evidence base for the treatment options that are currently available for facial BCCs.

## 2. Surgical Management

### 2.1. Standard Excision of Primary BCC with Predetermined Margins

Standard surgical excision is a highly effective treatment for primary BCC and historically has been the mostly common treatment option. BCCs are generally removed with a predetermined excision margin of 3-4 mm of normal skin. Especially on the face, grafts and flaps may be necessary to close the wound, rather than direct closure. 

A study of 2016 BCCs byBreuninger and Dietz[10], using horizontal sections to accurately detect BCC at any part of the surgical margin, found that excision of small (<10 mm diameter) lesions with a 2-mm peripheral surgical margin cleared 70%, margins of 3-mm cleared 84% and margins of 5-mm cleared 95% of all tumours. Morphoeic and large BCCs required wider surgical margins in order to maximize the chance of complete excision. For primary morphoeic lesions, the rate of complete excision was 66% for a 3-mm margin, 82% for 5-mm and >95% for 13–15 mm. 

Although little data exists on the correct deep surgical margin, excision through to the subcutaneous fat is generally advisable. Overall the 5-year recurrence rate after a simple excision of a BCC is reported as being between 4.1% [11] and 10.1% [12]. If the excision has been reported as histologically complete the recurrence rate is reported to be <2% [13]. This is due to sampling errors that occur as histological specimens are examined in a vertical plane. 

Basal cell carcinoma on the face may have a higher degree of subclincal spread than tumours arising elsewhere.Batra and Kelley [14] retrospectively analysed 1131 cases of Mohs' micrographic surgery on the face and found there to be higher rates of extensive subclinical spread on the nose, ear, eyelid, temple, and neck than the cheek. The nasal ala, nasal bridge, nasal tip, ear helix, and lower eyelid were all found to be locations of particularly high extensive spread. In addition, morpheaform BCCs were 2.3 times (*P* < .001), recurrent BCCs were 3.2 times (*P* < .001), and recurrent SCCs were 4.2 times (*P* = .01) more likely than nodular BCCs to exhibit extensive subclinical spread.

Generally the cosmetic outcome for standard surgical excision is felt to be good [15]. However, having to remove large lesions with adequate excision margins can be disfiguring as a result of loss of tissue, grafting, and subsequent scarring. Special attention must be paid to the location of the BCC on the face as there are many areas of functional and cosmetic importance for example the periocular, perioral, and perinasal areas. 

Various studies of incompletely excised BCCs suggest that not all recur and in a series of 74 patients Griffiths reports residual tumour in just 54% of re-excised tissue [16]. Studies with a 5 year followup have reported recurrence rates ranging from 21%–41% for patients following previous incomplete excisions [17, 18]. However, the study by Wilson et al. [17] of 140 patients, that reports a recurrence rate of 21% also reports that 31% of the cohort died of other causes during the (minimum 5-year) followup period and therefore the incidence may have been higher. 

When incomplete excision occurs on the face there is good evidence to support the need for re-excision. Boulinguez et al. [19] report a 24% chance of incompletely excised BCCs becoming more aggressive when they recur, with the likelihood being higher for perinasal, periocular and periauricular lesions. Although treatment should therefore be with early re-excision, there is a role for radiotherapy in special circumstances [20]. Recurrent tumours, especially on the face, are at high risk of further recurrence following surgical excision and require wider surgical margins. 

Generally standard surgical excision is considered a good treatment option for all BCCs arising on the face with 5-year recurrence rates of anything up to 10% providing adequate margins are taken. We would therefore recommend at least a 3-mm margin for standard surgical excision. While it would seem sensible to take larger margins at the sites where subclinical spread is known to be more extensive, these sites are all of great cosmetic and functional importance and therefore striking the correct balance is necessary while considering the option of Mohs' micrographic surgery as an alternative.

### 2.2. Mohs Micrographic Surgery

Mohs micrographic surgery (MMS) was first reported by an American physician and general surgeon, Dr. Mohs, in 1941 [21]. Excised tissue is frozen and sectioned horizontally. The entire margin can then be intraoperatively histologically examined. Further excision can then be performed if necessary from the specifically involved margin. MMS allows greater histological accuracy and increased tissue conservation. 

Studies and reviews have found 5-year cure rates of between 93.5% [22] and 100% [23] for primary tumours and 90% [22] to 96% [24] for recurrent disease.

A prospective randomised Dutch trial [11, 25] comparing MMS with standard surgical excision found recurrence rates to be comparatively lower after MMS for the treatment of both primary (pBCC) and recurrent BCCs (rBCC). For pBCCs rates were 2% and 3%, respectively, at 30 months and 2.5% and 4.1%, respectively, at 5 years. These differences were not statistically significant. In addition there was no significant difference in patient perception of cosmetic appearance following the procedure. For rBCCs, the rates were 0% and 3%, respectively, at 18 months and 2.4% and 12.1%, respectively, at 5 years. These differences were only statistically significant (*P* = .015) at 5 years. In addition, in the standard surgical group 30% of rBCCs were initially incompletely excised and required further surgery. This suggests that MMS should be the treatment of choice for facial rBCC on the basis of fewer recurrences. However, it is worth noting that in this study some patients who were randomised to standard surgical excision were moved to the MMS treatment group. Recurrent lesions retreated with the same modality have a greater risk of recurrence and in such cases MMS will offer the best chance of complete cure [26]. 

Even primary lesions need to be appropriately stratified to determine the optimal course of treatment [12, 27]. Histological subtype, location, size of the lesion, age and patient comorbidities are all important factors to consider. Guidelines suggest the following as being specific indications: Tumour site (especially central face, around the eyes, nose, lips, and ears), tumour size (any size, but especially >2 cm), histological subtype (especially morphoeic, infiltrative, micronodular, and basosquamous subtypes), poor clinical definition of tumour margins, recurrent lesions, and perineural or perivascular involvement [28].

MMS is more labour intensive and the cost of each procedure is significantly higher than for standard excision. However, in view of the reduced recurrence rate, MMS is cost effective treatment for appropriately selected cases. A recent study comparing Mohs' surgery to standard excision for facial and auricular nonmelanoma skin cancer found MMS to be more cost effective than standard surgical excision [29]. 

Mohs surgery provides the best chance of cure for all BCCs arising on the face with 5-year recurrence rates of anything up to 6.5%. However, due to time and cost limitations, it should be reserved for the treatment of high-risk primary or recurrent BCCs on the face.

### 2.3. Curettage with and without Cautery

Curettage is widely used in management of BCC. The tumour is scraped off with a curette and then the base and wound margin is often treated with electrocautery to control bleeding and destroy any residual tumour. This may be repeated. As excision margins are being destroyed it is advisable to confirm the diagnosis and determine the histological subtype with a preoperative biopsy, especially for facial lesions, unless a very confident clinical diagnosis can been made. 

For standard curettage and electrocautery recurrence rates have been reported to be between 7.7% [12] and 19% [30] at 5 years. Recurrence rates have been found to be much higher for facial lesions and recurrent disease [30, 31]. A prospective study of 69 re-excised BCC wounds immediately after curettage and electrocautery found residual tumour in 47% of head and neck wounds and 8.3% of trunk and limb wounds [32]. Curettage is very operator dependant; however, a retrospective study of curettage alone reported a 5-year cure rate of 96% for nonaggressive BCC, and tumours involving more than 50% of the deep edge of the specimen were found to have an increased risk of recurrence [33].

A randomised controlled trial comparing a double freeze thaw cycle of cryosurgery after curettage with standard excision for nonaggressive BCC of the head and neck reports recurrence rates of 17.6% and 8.2%, respectively [34]. However, there are other studies that report much lower recurrence rates than this. Lindemalm-Lundstam and Dalenbäck report a 1.5% recurrence rate following curettage and cryosurgery for head and neck BCCs after a median followup of 34-months [35].

Given the disproportionate amount of residual tumour on head and neck wounds and higher recurrence rates curettage and electrocautery is not considered first line treatment for BCCs on the face.

### 2.4. Cryosurgery

Cryosurgery involves the destruction of tissue using liquid nitrogen. Again, it is advisable to biopsy first to confirm the diagnosis and determine the histological subtype, especially for facial lesions. It is very operator dependant, and there are huge variations in practice. Data is therefore very inconsistent. Cryosurgery tends to be most useful in the treatment of low risk BCCs although good results have been reported following treatment of high risk lesions, either as sole treatment or in combination with curettage.

Recurrence rates are very variable, but when the lesion is carefully selected and in expert hands recurrence rates may be as low as 1% [36]. However, a nonrandomised study comparing cryosurgery (60 seconds freeze, 90 second thaw 2 × cycles) to radiotherapy found the 2-year recurrence rates to be 39% and 4%, respectively [37].

Cryosurgery wounds generally heal with minimal tissue contraction, resulting in good cosmetic results. However, a study comparing cryosurgery (20 seconds freeze, 60 seconds thaw 2 × cycles) to standard surgical excision for head and neck sBCC and small nBCCs found no significant difference in recurrence rates at 1-year but significantly worse cosmetic outcomes for those who had received cryosurgery [38]. 

Cryosurgery should not be first line in the management of facial BCCs due to the high risk or recurrence and potentially poorer cosmetic outcome.

### 2.5. Laser Ablation

Carbon dioxide laser ablation has been used in the treatment of BCCs. There are reports of use in combination with curettage for treatment of low-risk BCCs, but supportive data is generally lacking.

## 3. Non Surgical Management

### 3.1. Radiotherapy

Radiotherapy can be used to treat primary, recurrent or incompletely excised BCCs. It encompasses superficial X-ray and electron beam. Brachytherapy is used for contoured surfaces. The cure rates are over 90% for most skin lesions [12]. It may be used on tumours that occur in areas where surgery would either be technically difficult or would result in unacceptable amounts of tissue destruction. Radiotherapy therefore plays an important role in the management of head and neck BCCs. Tumours of the lower eyelid, inner canthus, lip, nose, and ear may be amenable to radiotherapy [39]. However, the upper eyelid is not an appropriate site for radiotherapy due to keratinization of the conjunctiva, lesions on the ear must be treated with caution due to the risk of damage to the underlying cartilage and the bridge of the nose is particularly susceptible to radionecrosis. Radiotherapy may be a good option for elderly patients with very large BCCs of the scalp. Radiotherapy is not appropriate for recurrent BCCs or patients with Gorlin's syndrome or with connective tissue disease. It tends not to be used in younger patients as skin cancers can arise from radiotherapy field scars and long term cosmetic results are poor. Side effects include radionecrosis, atrophy, and telangiectasia. Treatment in fractions over several visits may produce better cosmetic outcomes than a single fraction treatment. However, a daily regimen for a period of weeks may be a significant inconvenience to the patient as opposed to a single surgical treatment.

A randomised trial by Avril et al. [40] comparing radiotherapy to surgical excision for facial BCC of less than 4 cm found 4 year recurrence rates to be 7.3% and 0.7%, respectively. Cosmetic outcome also significantly favoured surgical excision at 4 years with 87% of patients assessing the surgical scar as good, compared to 69% after radiotherapy (*P* < .01) [41].

In addition, radiotherapy tends to be more expensive than any other form of treatment. A recent prospective study by Lear et al. in Canada [42] looked at the cost of MMS and radiotherapy for 49 BCCs. The authors found the cost of radiotherapy to be significantly greater and state direct costs of a “5-year cure" to be $952 (range $644–1,647) for MMS and $3,758 (range $3,564–4,675) for radiotherapy.

We believe radiotherapy is a good treatment option for facial BCCs located at difficult sites in patients who are not able to tolerate surgery.

### 3.2. Topical 5% Imiquimod Cream (Aldara)

Imiquimod is an immune response modifier. It acts by binding to toll-like receptor. This induces proinflammatory cytokine production and subsequent cytotoxic T cell mediated cell death. It is licensed for use in the treatment of sBCCs.

Vehicle-controlled studies in the treatment of small sBCC by Geisse et al. [43] have reported reasonable results. Twelve weeks following the 6 week treatment course the clearance rates were 82% (5x/week), 79% (7x/week) and 3% (vehicle only). Moderate to severe local site reactions occurred in 87% with erosions and ulceration in 36% and 22%, respectively. However, it is worth noting that facial BCCs were not included in this study. Schulze et al. [44] found similar clearance rates following a 6 weeks course of 7x/week topical imiquimod, with a 80% histological clearance compared to 6% for vehicle alone. However, long term clearance rates are lower. A prospective study of 182 patients who received topical imiquimod applied 5x/week for 6 weeks gave clearance rates of 69% at 5-years [45].

There is some data to suggest that imiquimod may be used in the treatment of nBCCs. A randomized dose-response study reported that 6 weeks after treatment with either a 6- or 12-week course of 7x/week imiquimod histological clearance rates were 71% and 76%, respectively [46]. A further randomized trial on nBCCs reported complete clinical clearance in 78% following 3x/week imiquimod. However, 8 weeks later excision revealed residual BCC in 13% of the patients considered to have shown complete clinical clearance [47].

In terms of studies specifically focusing on the treatment of facial BCCs with 5% imiquimod Vun and Siller [48] report a retrospective study of 19 lesions involving 12 patients. A once-daily treatment regimen for up to 9 weeks was used to treat both superficial and nodular basal cell carcinomas on the face. The authors report a clearance rate of 89.5% at an average of 39 months of followup. Another more recent study from 2010 [49] of fifteen patients with nodular BCCs on the eyelid, this time treated with 5% imiquimod once daily, 5 days/week for 6 weeks, reports that all tumors showed histopathological remission within 3 months of starting treatment, and sustained clinical remission was documented in each patient after 24–28 months' followup. The authors acknowledge that treatment tolerability was difficult in 7 patients with local effects being most problematic, but all symptoms disappeared when treatment ended and final aesthetic results were rated as excellent. They conclude that 5% imiquimod is a useful alternative to surgery in patients with periocular BCCs when other therapies have failed or are not possible.

Studies have also been done investigating the combination of curettage of nBCC prior to the use of topical imiquimod. Results have been variable with recurrence rates ranging from 6% [50] to 10% [51].

Effective treatment with imiquimod is dependent upon tissue penetration. sBCC may be more amenable to topical treatments as a result of their minimal depth of invasion. The increased depth of nodular tumours results in incomplete tumour penetration with the drug and hence lower clearance rates.

Imiquimod may be an alternative to surgery for patients with primary facial superficial BCCs, but long-term clearance is not as good as some of the other treatment modalities. It is not recommended for recurrent disease but is a good treatment option for elderly frail patients and patients who are not keen on surgical treatment.

### 3.3. Photodynamic Therapy (PDT)

Photodynamic therapy (PDT) involves the destruction of sensitised cells by an irradiating light source. A prodrug, either 5-aminolaevulinic acid (ALA) or methyl aminolaevulinic (MAL), is applied to the skin. This is converted intracellularly into protoporphyrin IX by the tumour cells. In the presence of intense red or blue light, a cytotoxic reaction occurs with reactive oxygen in the cell-membranes of tumour cells containing protoporphyrin IX and so the tumour cells are destroyed with sparing of uninvolved skin.

Superficial BCCs have been shown to achieve 87% clearance [52]. A prospective randomized study comparing PDT and standard surgical excision for the treatment of nBCCs found 5 years recurrence rates to be 14% and 4%, respectively; however, cosmesis was better for PDT with 87% of patients rating the cosmetic outcome as good compared to 54% for surgery [53]. PDT has also been compared with cryosurgery in the treatment of both sBCC and nBCC. Clinical recurrence rates at 12 months of 5% (PDT) and 13% (cryosurgery) were underestimates, as histology demonstrated residual BCC in 25% and 15% of cases, respectively [54].

Vinciullo et al. [55] reported 102 patients with sBCCs and nBCCs regarded as “difficult to treat” (defined as large and/or central facial lesions, or patients at increased risk of surgical complications) who received MAL-PDT treatment. Histologically confirmed clearance rates at 3 months were 93% (sBCC) and 82% (nBCC). The authors used a time-to-event approach to estimate sustained lesion clearance rates of 82% (sBCC) and 67% (nBCC) at 24 months.

Due to the clearance rates being lower than for surgical treatments, PDT is not generally recommended for management of nodular BCCs on the head or neck. While primary superficial BCCs on the face may be amenable to treatment is not recommended for recurrent disease.

### 3.4. Topical 5-Fluorouracil 5% (Efudex)

5-fluorouracil is a fluorinated pyrimidine that blocks the methylation reaction of deoxyuridylic acid to thymidylic acid and in doing so destabilises DNA. It is sometimes used to treat small, superficial BCCs and should only be used on low risk sites. It therefore is not recommended in the management of facial BCCs.

## 4. Conclusion

We have discussed a number of the different treatment options available for BCCs. However, lesions on the face are considered high-risk, and therefore, some of the treatment modalities that would otherwise be considered may not always be appropriate. It is often necessary to a prior histological diagnosis, especially if a destructive treatment is being considered. Mohs micrographic surgery remains the gold standard, but it is not feasible for it to be offered to all. Standard surgical excision gives good results in most cases. Radiotherapy may be considered for patients where surgery is not an option. Other treatment options include curettage and electrocautery, PDT, laser, topical imiquimod and cryosurgery; however, in the majority of cases these treatments should not be first line due to the risk of recurrence but may be a good option in the elderly population. In addition, it is important to consider patient choice, feasibility, side effects, and cosmetic outcome when planning management.
